# Phytochemical Comparison of Medicinal Cannabis Extracts and Study of Their CYP-Mediated Interactions with Coumarinic Oral Anticoagulants

**DOI:** 10.1159/000528465

**Published:** 2023-02-08

**Authors:** Andrea Treyer, Jakob K. Reinhardt, Daniela Elisabeth Eigenmann, Mouhssin Oufir, Matthias Hamburger

**Affiliations:** ^a^Division of Pharmaceutical Biology, University of Basel, Basel, Switzerland; ^b^Bahnhof Apotheke Langnau AG, Langnau im Emmental, Switzerland; ^c^Oncodesign SA, Villebon-sur-Yvette, France

**Keywords:** Cannabinoids, Chemotypes, Vitamin K antagonists, CYP-mediated herb-drug interactions, Coumarinic oral anticoagulants, In vitro CYP inhibition assays, LC-MS/MS

## Abstract

**Introduction:**

Treatment with cannabis extracts for a variety of diseases has gained popularity. However, differences in herb-drug interaction potential of extracts from different plant sources are poorly understood. In this study, we provide a characterization of cannabis extracts prepared from four cannabis chemotypes and an in vitro assessment of their Cytochrome P450 (CYP)-mediated herb-drug interaction profiles.

**Methods:**

Plant extracts were either commercially obtained or prepared using ethanol as solvent, followed by overnight decarboxylation in a reflux condenser system. The extracts were characterized for their cannabinoid content using NMR and HPLC-PDA-ELSD-ESIMS. CYP inhibition studies with the cannabis extracts and pure cannabinoids (tetrahydrocannabinol [THC] and cannabidiol [CBD]) were performed using pooled, mixed gender human liver microsomes. Tolbutamide and testosterone were used as specific substrates to assess the inhibitory potential of the extracts on CYP2C9 and CYP3A4, and the coumarinic oral anticoagulants warfarin, phenprocoumon, and acenocoumarol were studied as model compounds since in vivo herb-drug interactions have previously been reported for this compound class.

**Results:**

In accordance with the plant chemotypes, two extracts were rich in THC and CBD (at different proportions); one extract contained mostly CBD and the other mostly cannabigerol (CBG). Residual amounts of the corresponding acids were found in all extracts. The extracts with a single major cannabinoid (CBD or CBG) inhibited CYP2C9- and CYP3A4-mediated metabolism stronger than the extracts containing both major cannabinoids (THC and CBD). The inhibition of CYP3A4 and CYP2C9 by the extract containing mostly CBD was comparable to their inhibition by pure CBD. In contrast, the inhibitory potency of extracts containing both THC and CBD did not correspond to the combined inhibitory potency of pure THC and CBD. Although being structural analogs, the three coumarin derivatives displayed major differences in their herb-drug interaction profiles with the cannabis extracts and the pure cannabinoids.

**Conclusion:**

Despite the fact that cannabinoids are the major components in ethanolic, decarboxylated cannabis extracts, it is difficult to foresee their herb-drug interaction profiles. Our in vitro data and the literature-based evidence on in vivo interactions indicate that cannabis extracts should be used cautiously when co-administered with drugs exhibiting a narrow therapeutic window, such as coumarinic anticoagulants, regardless of the cannabis chemotype used for extract preparation.

## Introduction

Cannabis is one of the earliest psychoactive plants that were cultivated by *Homo sapiens* for fibers but also for recreational and pharmacological use [1, 2]. It is a dioecious plant originating from Central Asia that grows today almost around the world [3, 4]. More than 120 cannabinoids have been identified in cannabis up to date, with Δ9-tetrahydrocannabinol (THC) and cannabidiol (CBD) being the most abundant ones [5, 6]. Cannabinoids are usually present in the plant as their acids. Upon heating or drying, they are transformed into their neutral forms via a non-enzymatic thermal decarboxylation [7]. Pharmacological activity is mainly attributed to the decarboxylated forms. However, there is emerging evidence that acidic precursors of the cannabinoids also contribute to the pharmacological effects [8].

Cannabis and preparations thereof have traditionally been used as medicinal products to treat a variety of diseases. Around 1900, cannabis formulations were frequently utilized in Europe to medicate muscular spasms, migraine, and sleep disturbances, among others [9]. However, from the mid-20th century onward, their use became severely limited due to various reasons. Among others, difficulties existed in ensuring quality and efficacy of cannabis-based preparations, and they were thus increasingly replaced in the medicinal practice by novel, well-characterized, and mostly synthetic drugs. Furthermore, importation of Indian cannabis to Europe was hampered due to the World Wars, and economic problems emerged in the production of cannabis formulations. Finally, the implementation of increasingly restrictive laws and legal sanctions culminated in the worldwide ban of cannabis in 1961 [10].

The discovery of the endocannabinoid system in the 1990s provided the pharmacological basis for an understanding of how THC elicits its psychoactive effects, sparked renewed interest in the almost forgotten medicinal plant, and led to numerous research projects, which are still increasing today [11, 12, 13, 14]. In several countries, patients have nowadays again legal access to medicinal cannabis preparations for oral use, such as oils, solutions and tinctures, or are even allowed to use dried cannabis flowers for inhalation.

While preparations rich in THC are primarily used to treat chronic pain, muscular spasms, emesis, and nausea, CBD-based medicinal products are mainly prescribed for the treatment of refractory epilepsy, psychiatric disorders such as anxiety and psychosis, and inflammatory pain [15, 16]. As the intake of cannabis formulations by polymedicated patients is increasing, the risk for potential drug-drug and herb-drug interactions needs to be taken into consideration.

Recent studies showed an inhibitory potential of isolated cannabinoids toward metabolizing enzymes (Cytochrome P450 (CYP), Uridine 5′-diphospho (UDP)-glucuronosyltransferases, and carboxylesterases) [17, 18, 19, 20, 21, 22, 23]. However, while these studies provided information on drug interactions with pure cannabinoids, little is known about herb-drug interactions with full plant extracts containing varying amounts of the major cannabinoids THC and CBD and other therapeutically promising cannabinoids, such as cannabigerol (CBG) [24]. The aim of this study was thus to investigate the inhibitory potency of four selected medical cannabis extracts with different cannabinoid profiles. Some of these extracts are commercially available in Switzerland. Prior to their in vitro assessment for herb-drug interactions, all extracts were characterized for their cannabinoid content using NMR and LC-UV-MS. Potential metabolic herb-drug interactions of two of the selected cannabis extracts were studied in more detail, using human liver microsomes (HLM) and the coumarinic anticoagulants warfarin, phenprocoumon, and acenocoumarol. These drugs are widely used for blood thinning, and, given their narrow therapeutic window, CYP-mediated interactions pose a risk in clinical practice.

## Materials and Methods

### Chemicals and Reagents

All solvents and reference compounds were obtained from Sigma-Aldrich or Toronto Research Chemicals in their purest available form (≥98%) unless otherwise stated. Pure CBD (Trigal Pharma GmbH, Wien, Austria, ≥98% purity) and THC (THC Pharm GmbH The Health Concept, Frankfurt, Germany, ≥98% purity) were kindly provided by Bahnhof Apotheke Langnau AG, in the case of THC under a permit issued by the Federal Office of Public Health (FOPH) of Switzerland (permit No. 2019/010338).

### Preparation of Cannabis Extracts from Dry Plant Material

Dry plant material was obtained from Cannapharm AG, Burgdorf, Switzerland. 50 g of dried plant of chemotypes *C25X* (containing mainly CBD; THC and CBG <1%) and *CBG7* (containing mainly CBG; THC and CBD <1%) was ground using an M20 universal mill (IKA) and stirred with 250 g (*C25X*) or 200 g (*CBG7*) ethanol (75% m/m) for 2 h. The residue was separated from the extraction solvent by means of vacuum filtration, and the extract was decarboxylated for 12 h at 80°C using a reflux condenser system. The extracts were stored at 4°C before analysis. The extract from chemotype C25X was designated “*extract CBD*,” and the extract from chemotype CBG7 “*extract CBG*.”

Commercially available extracts with standardized THC and CBD contents were obtained from Cannapharm AG, Burgdorf, Switzerland (designated “*extract THC-CBD*,” ratio THC:CBD 1:2), and GW Pharma Ltd., Cambridge, GB (*Sativex*®, standardized mixture of two extracts obtained from a THC and a CBD chemotype of *Cannabis sativa*, also known as “*extract nabiximols*,” ratio THC:CBD 1:1). These two extracts were obtained under a permit issued by the Federal Office of Public Health (FOPH) of Switzerland, permit No. 2019/010338.

### Characterization of Plant Extracts

*HPLC-PDA-ELSD-ESIMS* data were recorded on an 8030 triple quadrupole ESIMS system connected to an HPLC system consisting of a degasser, binary high-pressure mixing pump, autosampler, column oven, and photodiode array detector (all Shimadzu). An Alltech 3300 ELSD detector was connected between the photodiode array and the MS detector via a T splitter. HPLC separations were carried out on a Waters SunFire^TM^ C_18_ column (3.5 μm, 3.0 × 150 mm i.d.) equipped with a guard column (3 × 10 mm i.d). Mobile phase: water + 0.1% formic acid (A), acetonitrile + 0.1% formic acid (B); 70% B (0–2 min), 70–77% B (2–20 min), 77–100% B (20–25 min), 100% B (25–29 min). Extracts were diluted 40-fold, and the injection volume was 2 μL. Analytical standards were used for identification and quantification of THC, CBD, and CBG. Serial dilutions with eight concentrations ranging from 7.8 μg/mL to 1 mg/mL were prepared in triplicate.

For *NMR analysis*, 50 µL of each extract was dried under a flow of N_2_. The residue was dissolved in 500 µL CDCl_3_, and spectra were recorded at 23°C on an Avance Ultrashield NMR Spectrometer (Bruker) operating at 500 MHz for ^1^H. ^1^H, HSQC-DEPT, and HMBC spectra were recorded using a BBO-probe head. To enable quantitative evaluation of the integrals, ^1^H spectra were recorded with an extended relaxation delay D1 of 63 s. ACD/Spectrus Processor software (ACD/Labs) was used for data evaluation. Relative quantification of cannabinoids was performed by using the integrals of prominent signals of each compound. For an intuitive comparison, the relative contents of other cannabinoids are then given in percent relative to the main cannabinoid. As the CH_2_−1″ signal of the pentyl side chain of cannabinoid acids is typically found in the region between δ_H_ 2.75 and 2.90 ppm without the interference from other cannabinoids [25, 26], this region was integrated and compared to the sum of THC, CBD, and CBG identified in the sample. This allowed to estimate the amount of cannabinoid acids left in the extract after decarboxylation.

#### CYP Inhibition Assays

Pooled HLM (Bioreclamation, 150 donors, mixed gender) were incubated with cannabis extracts or pure THC or CBD as reference (range of 0.1 nM–100 µM). Sulfaphenazole and ketoconazole (range of 0.1 nM–100 µM) were used as CYP2C9 and CYP3A4/5 isoform-specific inhibitors, respectively (control experiments are presented in online suppl. material 3; for all online suppl. material, see www.karger.com/doi/10.1159/000528465). The substrates warfarin (10 µM), acenocoumarol (25 µM), and phenprocoumon (10 µM) were added at concentrations approximating their reported K_m_ values [27]. Tolbutamide (100 µM) and testosterone (75 µM) were used as CYP2C9 and CYP3A4/5 isoform-specific substrates, respectively, at concentrations commonly used in screening assays (control experiments are presented in online suppl. material 3).

Briefly, 96-well incubation plates were prepared by adding inhibitors, substrates, and extracts (all dissolved in ethanol) to the respective wells. The 96-well plates were then placed on a thermoshaker (37°C, 500 rpm) until full evaporation of the solvent. This evaporation step was found to be crucial, as ethanol has been demonstrated to have particularly strong inhibitory effects on CYP2C9 at concentrations as low as 0.3% [28]. HLM were thawed on ice, diluted in 100 mM phosphate buffer (pH 7.4) (final concentration 0.2 mg/mL), and added to the incubation plate that was prepared with the inhibitors and substrates. For preincubation, the plates were shaken at 500 rpm on an orbital shaker for 15 min at 37°C. The reaction was started by addition of NADPH (diluted in 100 mM phosphate buffer pH 7.4, 10x concentrated, final concentration 1 mM) and stopped after 10 min (testosterone), 15 min (warfarin, phenprocoumon, acenocoumarol), or 20 min (tolbutamide) by transferring the samples to a plate containing an equal volume of ice-cold acetonitrile containing the internal standard. The formation of hydroxylated metabolites was followed using UHPLC-MS/MS quantification with electrospray ionization in positive mode, as described in online supplementary material 2.

#### Statistical Analysis

GraphPad Prism, version 9.0.1, was used for fitting of IC_50_ curves (inhibitor vs. normalized response, variable slope).

## Results

### Phytochemical Comparison

**Fig. 1:**
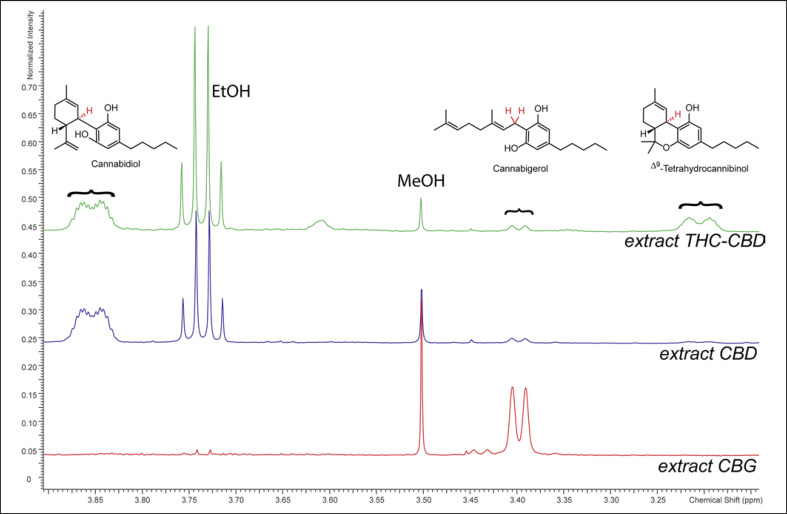
^1^H NMR spectra between 3.15 and 3.90 ppm of the extracts. The characteristic signals from CBD, CBG, and THC are marked. The spectrum of extract nabiximols is not shown due to the interference of propylene glycol in this spectral region.

The composition of the four extracts *extract THC-CBD*, *extract CBD*, *extract CBG*, and *extract nabiximols* was first analyzed by NMR. Sections of the ^1^H spectra containing characteristic signals of THC, CBD, and CBG are shown in Figure 1. Full ^1^H NMR spectra with integrals used for relative quantification by QNMR are presented in online suppl. material 1 (online suppl. Fig. 1–4), and ^1^H-^1^H COSY, HSQC-DEPT, and HMBC spectra are provided in online suppl. material 1 (online suppl. Fig. 5–13). In all extracts, traces of ethanol were detected but did not hinder analysis of the cannabinoid composition.

In *extract THC-CBD*, THC and CBD were identified as main components present in a ratio of 1:2. The relative content of CBG was estimated based on its signal at δ_H_ 3.40 ppm (H_2_−1, δ_C_ 22.1 ppm) to be present at approx. 16% relative to THC. Another cannabinoid was assigned in comparison to literature as cannabichromene (CBC; H-1′ and H-2′, δ_H_ 6.62 and 5.50 ppm) [29]. CBC was quantified using the H-1′ signal (δ_H_ 6.62 ppm) to be present at approx. 12% relative to THC. From integrating the region between δ_H_ 2.75 and 2.90 ppm containing the CH_2_−1″ signals of cannabinoid acids, a maximum of 23% of all identified cannabinoids were present as their acids in *extract THC-CBD.*

In *extract CBD*, CBD was identified as the major cannabinoid. For relative quantification, the signal of H-1 (δ_H_ 3.85 ppm, δ_C_ 37.2 ppm in HMBC spectrum), as well as the two signals originating from the vinyl group (H_2_−8, δ_H_ 4.57 and 4.67 ppm, δ_C_ 110.8 ppm) were used. THC was quantified using the H-1 signal (δ_H_ 3.20 ppm) to be present at approx. 10% of the amount of CBD. CBG was estimated based on its H_2_−1 signal (δ_H_ 3.40 ppm) to be present at about 5% relative to CBD. CBC was quantified using the H-1′ signal (δ_H_ 6.62 ppm) to be present 8% relative to CBD. A maximum of 13% of all identified cannabinoids in *extract CBD* were present as their acids.

In *extract CBG*, CBG was identified as the major component. The signal of H_2_−1 (δ_H_ 3.40 ppm, δ_C_ 22.2 ppm) and the two signals originating from the two olefinic protons H-2 and H-6 (δ_H_ 5.28 ppm and 5.06 ppm; δ_C_ 121.6 and 123.7 ppm) were used for relative quantification. No THC was found in this extract. CBD was quantified using the signals of H_2_−8 (δ_H_ 4.57 and 4.67), and H-2 (5.58 ppm; δ_C_ 125.4 ppm), to be present at about 4% relative to CBG. CBC was quantified using the H-1′ signal (δ_H_ 6.62 ppm) to be present in 2% the amount of CBD. In *extract CBG*, a maximum of 23% of all identified cannabinoids were present as their acids.

In the NMR spectrum of *extract nabiximols*, the region between 3.2 and 4.0 ppm overlapped with signals from contained propylene glycol, but signals for THC, CBD, and CBC could be identified. Two signals originating from the vinyl group of CBD (H_2_−8, δ_H_ 4.57 and 4.67 ppm) were clearly visible, and thus other integrals were compared to them. THC was quantified using the H-1 signal (δ_H_ 3.20 ppm) and found to be present at approx. 150% relative to CBD. Due to a slight overlap with an adjacent signal, this value has to be considered as an approximation. The quantity of CBC was 6% relative to CBD, while CBG was not detected in the NMR spectrum of this extract. A maximum of 12% of identified cannabinoids were present as their acids in *extract nabiximols*.”

**Fig. 2:**
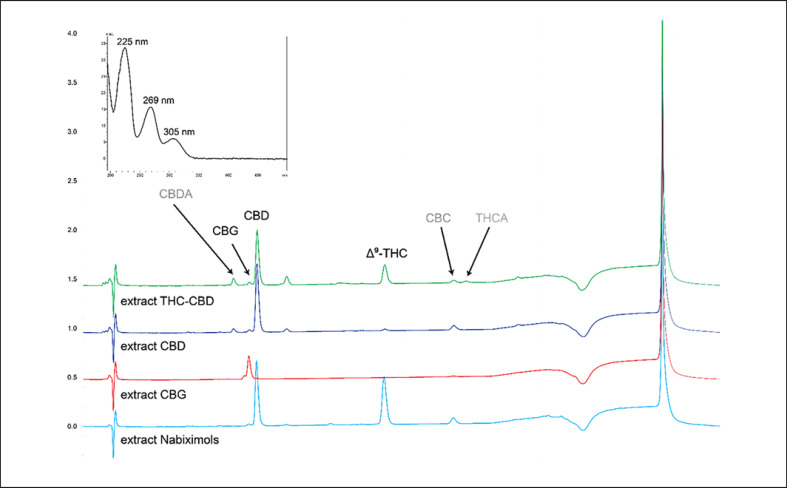
Extracted HPLC-UV chromatograms of cannabis extracts at 225 nm. Compounds labeled in black were identified via a comparison with analytical standards (CBD, CBG, THC). Compounds labeled in gray (cannabidiolic acid [CBDA], cannabichromene [CBC], and tetrahydrocannabinolic acid [THCA]) were tentatively assigned based on MS data, UV data, and by a comparison with reported HPLC data [30]. The extracted UV spectrum for CBDA is shown.

Cannabinoids are readily detected at 225 nm in the HPLC-UV chromatogram (Fig. 2). In addition to the peaks of CBD, CBG, and THC, two additional small peaks eluting at t_R_ 8.26 and 21.03 min were observed. In ESIMS, both gave an m/z 559 in positive mode and m/z 557 in negative mode, corresponding to the [M+H]^+^ and [M−H]^−^ ions of cannabidiolic acid (CBDA) and tetrahydrocannabinolic acid (THCA). Thus, they were tentatively assigned based on MS and UV spectra (λ_max_ at 225, 269 and 305 nm, Fig. 2) as well as previously reported chromatographic mobility under similar conditions [30, 31]. The peak of CBC at t_R_ 20.30 was assigned based on its m/z of 315 in positive mode in ESIMS. Finally, THC, CBD, and CBG were quantified individually with the aid of calibration curves of reference compounds. Then, the total cannabinoid content was calculated as the sum of these three major cannabinoids (Table 1).

### CYP Inhibition Assays

**Fig. 3:**
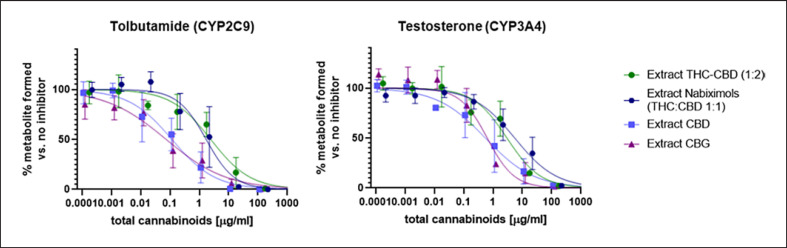
Inhibition curves of the four extracts (based on total cannabinoid content) versus specific CYP substrates.

The inhibition of CYP isoenzymes by differing cannabinoid compositions of the extracts was studied. For the specific substrates tolbutamide (CYP2C9) and testosterone (CYP3A4), the inhibition of formation of hydroxylated metabolites is shown in Figure 3, and control experiments using specific CYP inhibitors are presented in online supplementary material 3. Large differences in inhibitory potency were observed between the different extracts. *Extract CBD* and *extract CBG*, which both contained a single major cannabinoid, showed approx. 10-fold lower IC_50_ values for CYP2C9 and 5-fold lower IC_50_ values for CYP3A4 inhibition than the two extracts containing a combination of two cannabinoids (THC and CBD) (Fig. 3; online suppl. material 3).

**Fig. 4:**
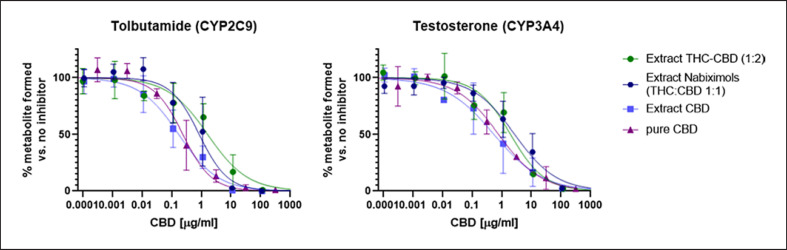
Inhibition curves of the three extracts (based on CBD content) and pure CBD versus specific CYP substrates.

**Fig. 5:**
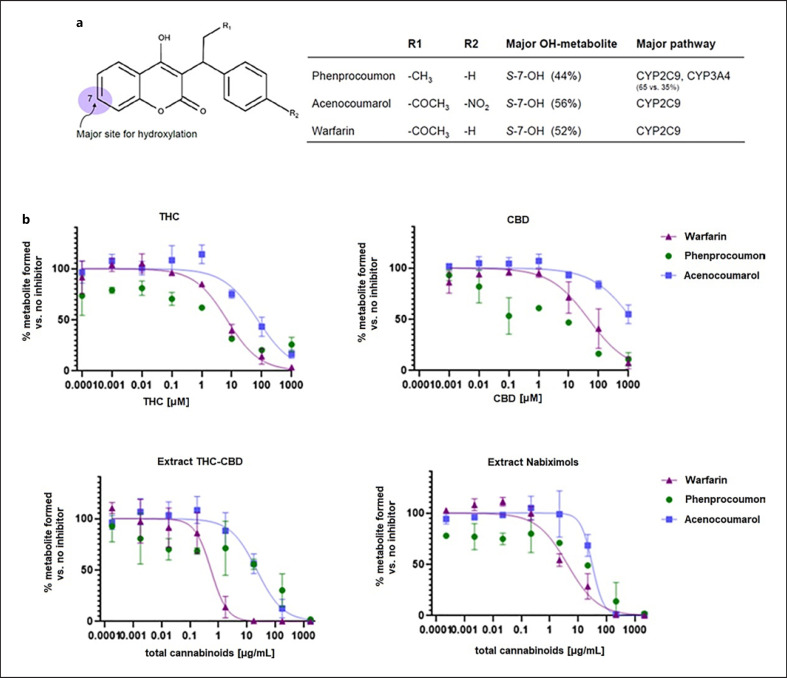
**a** Major metabolic pathways and site of hydroxylation of vitamin K antagonists [32, 34, 35]. **b** Inhibition curves of pure THC, pure CBD, and the two commercially available extracts (*extract THC-CBD* and *extract nabiximols*) against warfarin (triangles), phenprocoumon (circles), and acenocoumarol (squares).

*Extract CBD* compared well to pure CBD (Fig. 4), whereas the presence of THC in *extract THC-CBD* and *extract nabiximols* weakened the CYP inhibition, regardless of the THC:CBD ratio in the extracts and despite the fact that THC may act as a potent CYP inhibitor itself (IC_50_ of 0.081 µM and 23.6 µM for CYP2C9 and CYP3A4, respectively; online suppl. material 3). Finally, the inhibitory potency of pure THC, CBD, and the two commercially available extracts *extract THC-CBD* and *extract nabiximols* toward metabolism of warfarin (major pathway via CYP2C9), phenprocoumon (major pathway via CYP2C9 and CYP3A4), and acenocoumarol (major pathway via CYP2C9) was assessed. For all three compounds, the 7-hydroxylated form is the major metabolite (shown in Fig. 5a), but they also undergo different metabolic pathways. Generally, the hydroxylation of warfarin was inhibited at approximately 10-fold lower cannabinoid concentrations than formation of hydroxylated acenocoumarol. The IC_50_ curve of hydroxylated phenprocoumon did not follow a classical sigmoidal curve (shown in Fig. 5b), possibly due to its more complex metabolic pathway and formation of multiple metabolites (six hydroxylated metabolites have been described in literature) [32, 33]. Interestingly, based on total cannabinoid content, the two extracts inhibited the metabolism at lower concentrations than what would have been expected based on combination of the two pure compounds (shown in Fig. 5b) and based on the learnings from the specific substrates tolbutamide and testosterone (shown in Fig. 4). This indicated that the combined effect of cannabinoids in extracts on CYP inhibition cannot be foreseen for drugs with multiple metabolic clearance pathways.

## Discussion

The taxonomic classification of the genus *Cannabis* is still debated. While some researchers propose three cannabis species (*C. sativa*, *C. indica*, and *C. ruderalis*) [36], the monotypic concept of *Cannabis sativa* having several subspecies such as *C. sativa* subsp. *indica* is preferred by most taxonomists [37, 38]. A classification into five chemotypes based on the THCA/CBDA ratio has furthermore been recognized [39]. Chemotype I, usually intended for recreational use, is characterized by a high THCA/CBDA ratio (>>1). In chemotype II, the THCA/CBDA ratio is intermediate (0.5–2.0), and plants of chemotype III have a low THCA/CBDA ratio (<<1) [39]. Chemotype IV is characterized by a low content of both THCA and CBDA but a high content of CBGA. Chemotype V is devoid of most cannabinoids and used mostly for fiber production [39]. In this study, four cannabis extracts produced from different plant chemotypes were characterized.

The plant material studied here belongs to the chemotypes II (*extract THC-CBD*), III (*extract CBD*), and IV (*extract CBG*). The *extract nabiximols* is a mixture of separate extracts from chemotypes I and III. Since the commercially available cannabis extracts used in this study were standardized for the neutral forms of the cannabinoids (and not their acids), the extracts that were prepared in-house (*extract CBD* and *extract CBG*) were decarboxylated by a heat treatment after the extraction to allow for comparison [40].

Recent studies investigated the inhibitory potency of isolated cannabinoids toward CYP enzymes. In particular, Yamaori et al. [17, 41, 42, 43] studied the effect of pure cannabinoids and some chemical substructures found in cannabinoids (e.g., resorcinol, orcinol, D-limonene, and olivetol) toward several CYP enzymes (CYP1A2, CYP2B6, CYP2D6, CYP2C9, CYP2C19, and CYP3A4)[41, 42, 43]. In these studies, THC and CBD showed IC_50_ values in the low micromolar range, although the inhibitory potency varied depending on the enzyme source and the substrates used [17, 19, 41, 42, 43]. Bansal et al. [20] further determined bound and unbound IC_50_ values of THC and CBD toward CYP1A2, CYP3A4, CYP2C9, CYP2C19, and CYP2D6. With a fraction unbound of 0.05 and 0.12, respectively, at 0.5 mg/mL protein concentration, the binding-corrected inhibitory potency was in the nanomolar range. Using a mechanistic static model, a moderate to strong pharmacokinetic interaction potential was predicted between orally administered CBD and THC with drugs that are extensively metabolized by CYP1A2, CYP2B9, CYP2C9, CYP2C19, CYP2D6, CYP3A and CYP1A2, CYP2C9, CYP3A, respectively [18, 20].

A review focusing on clinical studies suggested that care needs to be taken regarding oral administration of cannabinoid-containing medicines [18]. The review provides a list of as many as 57 medications with a narrow therapeutic index and a total of 139 medications that can potentially be impacted by concomitant use of cannabinoids. Among these, the coumarinic oral anticoagulants used in this study (warfarin, phenprocoumon, and acenocoumarol) are prominent examples, and clinically relevant case-studies with impact on the target international normalized ratio (INR) have been reported for warfarin [44, 45].

Despite these recent advancements in the field, little is known about the combined effect on CYP inhibition of extracts with differing cannabinoid compositions. We therefore conducted in vitro experiments with cannabis extracts prepared from different plant chemotypes and characterized the composition of the extracts by NMR and HPLC-UV-ESI-MS. *Extract THC-CBD* and *extract nabiximols* were mainly composed of THC and CBD (at relative concentrations of 1:2 and 1:1, respectively) and minor amounts of CBG and CBC. *Extract CBD* contained THC, CBG, and CBC as minor cannabinoids, while *extract CBG* contained small amounts of CBD and CBC but no THC.

Regardless of the plant chemotype and cannabinoid composition, all extracts strongly inhibited CYP3A4 and CYP2C9 in vitro (Fig. 3). Extracts from chemotypes with one predominant cannabinoid (*extract CBD* and *extract CBG*) showed lower IC_50_ values than extracts containing a mixture of THC and CBD (*extract THC-CBD* and *extract nabiximols*), regardless of the THC/CBD ratio in the mixtures. The inhibitory potency of *extract CBD* was comparable to that of pure CBD. Pure CBD inhibited both CYP2C9 and CYP3A4 at similar levels (0.76 vs. 2.5 µM), while THC showed higher potency toward CYP2C9 (0.081 vs. 23.6 µM). This finding is in line with previously published data [20].

For the vitamin K antagonists, a stronger inhibition was observed with the extracts than with the pure compounds (Fig. 5b). Also, the formation of hydroxylated metabolites of warfarin, acenocoumarol, and phenprocoumon was affected at different cannabinoid concentrations, despite the fact that they are structural analogs and that CYP2C9 had been identified as the major isoenzyme catalyzing their hydroxylation (shown in Fig. 5a) [27]. However, other CYP enzymes, such as CYP1A2, CYP2C8, CYP2C19, and CYP3A4, are involved in hydroxylation at different positions of the coumarin scaffold, all of which are inhibited by cannabinoids to varying degrees [20, 27, 34]. Thus, an assessment of the interaction potential of cannabinoid mixtures remains extremely challenging, and further studies, e.g., by using recombinant CYP enzymes and determination of K_m_ and V_max_ for each isoform, are warranted.

For both pure cannabinoids and the different extracts, warfarin had a less favorable inhibition profile than acenocoumarol (Fig. 5b). This possibly explains why several case studies on clinically significant interactions were reported for warfarin but not for acenocoumarol [44, 45]. However, in vitro findings cannot directly be extrapolated to the in vivo situation. In vivo, additional factors need to be considered, such as the high plasma protein binding of cannabinoids which can result in a displacement of highly bound drugs, such as warfarin [18, 32, 46]. Moreover, the lipophilic nature of cannabinoids results in tissue accumulation and subsequent redistribution into body fat [46, 47] and, as a consequence, to slow clearance and long half-lives [46, 48, 49]. Some cannabinoid metabolites in humans (e.g., 11-OH-THC and 11-nor-9-COOH-THC) have been shown to inhibit various CYP enzymes [20]. Additionally, CYP1A2, which is induced in smoking individuals, is also known to have a large inter-individual variability [18]. Finally, there are reports of in vivo CYP-induction by cannabinoids after long-term use [50]. Repeated oral administration of low doses of cannabis extracts may therefore result in an even higher risk for drug-herb interaction in vivo than predicted in vitro, despite their low oral bioavailability [37].

Our in vitro results indicate that extracts from cannabis chemotypes with a single dominant cannabinoid (CBD or CBG) may have a stronger inhibitory potency than extracts containing a mixture of THC and CBD. The overall inhibitory potency of extracts could not be extrapolated from the inhibitory potencies of pure THC and pure CBD, and the combined effect of THC and CBD in an extract pointed at different directions depending on the substrates (higher IC_50_ for tolbutamide and testosterone, lower IC_50_ for coumarinic anticoagulants). Taken together, our in vitro data and published evidence on the in vitro and in vivo interaction potential indicate that oral cannabis extracts should be used cautiously when combined with drugs possessing a narrow therapeutic window, such as coumarinic oral anticoagulants. This applies regardless of the cannabis chemotype used for extract preparation. This precaution may be especially important when oral cannabis extracts are used in an elderly and, thus, often polymedicated population.

## Statement of Ethics

An ethics statement was not required for this study type; no human or animal subjects were used.

## Conflict of Interest Statement

Daniela Elisabeth Eigenmann works as pharmacist in the Bahnhof Apotheke Langnau AG, where medical cannabis-based preparations (among others, *extract THC-CBD* and *extract nabiximols*) are distributed to patients upon medical prescription. She is a board member of the Swiss Society of Cannabis in Medicine. She had no role in data analysis but edited and critically reviewed the manuscript for important intellectual content. All other authors have no conflicts of interest to declare.

## Funding Sources

This study was funded in part by Bahnhof Apotheke Langnau AG, Langnau, Switzerland (Manfred Fankhauser) and Cannapharm AG, Burgdorf, Switzerland (Markus Lüdi).

## Author Contributions

Andrea Treyer contributed in conceptualization and study design and carried out the in vitro HLM experiments and LC-MS/MS analysis. Jakob K. Reinhardt produced plant extracts and carried out NMR and HPLC-PDA-ESI-MS experiments. Andrea Treyer and Jakob K. Reinhardt wrote their respective parts of the manuscript. Daniela Elisabeth Eigenmann edited and critically reviewed the manuscript for important intellectual content. Mouhssin Oufir and Matthias Hamburger contributed to the conceptualization and study design and critically reviewed the manuscript. All authors read and approved the final manuscript.

## Data Availability Statement

Raw data are stored in archives at the University of Basel. All relevant data are included in the publication/online supplementary material. Further inquiries can be directed to the corresponding author.

## Supplementary Material

Supplementary data

## Figures and Tables

**Table 1:** Cannabinoid content in extracts, with relative amounts determined by NMR, and quantitative data obtained by HPLC-UV-ESIMS analysis

Extract	NMR THC:CBD:CBG ratio signal δ_H_	HPLC-UV-ESIMS			Total cannabinoid content, mg/mL
		THC, mg/mL	CBD, mg/mL	CBG, mg/mL	
Extract THC-CBD	**1:2**:0.16	**10.8±1.9**	**25.2±4.6**	<1	36.0
Extract CBD	0.09:**1**:0.05	<1	**28.1±2.9**	<1	28.1
Extract CBG	0.00:0.04:**1**	n.d.	n.d.	**8.9±1.8**	8.9
Extract nabiximols	**˜1.5:1**:n.d.*	**28.2±0.8**	**30.9±0.6**	<1	59.1

Data are presented as mean ± SD of two independent measurements. n.d., not detected.

* Uncertainty due to interference with propylene glycol present in formulation.
