# Supplementary material for: Phytochemical Comparison of Medicinal Cannabis Extracts and Study of Their CYP-Mediated Interactions with Coumarinic Oral Anticoagulants
Source: Med Cannabis Cannabinoids. 2023 Feb 8;6(1):21–31. doi: 10.1159/000528465 (PMC9940649; doi:10.1159/000528465)
Supplement: Supplementary file 1 — Supplementary data [file mca-0006-0021-s01.pdf]

Phytochemical comparison of medicinal cannabis extracts and study of their CYP-mediated interactions with coumarinic oral anticoagulants

Treyer Andrea, Reinhardt Jakob K., Eigenmann Daniela Elisabeth, Oufir Mouhssin, Hamburger Matthias

Supplementary Material 1: NMR spectra

Supp. Fig. 1. ^1H NMR spectrum of extract nabiximols (500 MHz, $CDCl_3$) with integrals used for relative quantification

Supp. Fig. 2. ^1H NMR spectrum of extract THC-CBD (500 MHz, $CDCl_3$) with integrals used for relative quantification

Supp. Fig. 3. ^1H NMR spectrum of extract CBD (500 MHz, $CDCl_3$) with integrals used for relative quantification

Supp. Fig. 4. ^1H NMR spectrum of extract CBG (500 MHz, $CDCl_3$) with integrals used for relative quantification

Supp. Fig. 5. ^1H-^1H COSY NMR spectrum of extract THC-CBD (500 MHz, $CDCl_3$)

Supp. Fig. 6. HSQC-DEPT NMR spectrum of extract THC-CBD (500 MHz, $CDCl_3$)

Supp. Fig. 7. HMBC NMR spectrum of the extract THC-CBD (500 MHz, $CDCl_3$)

Supp. Fig. 8. ^1H-^1H COSY NMR spectrum of extract CBD (500 MHz, $CDCl_3$)

Supp. Fig. 9. HSQC-DEPT NMR spectrum of extract CBD (500 MHz, $CDCl_3$)

Supp. Fig. 10. HMBC NMR spectrum of extract CBG (500 MHz, $CDCl_3$)

Supp. Fig. 11. ^1H-^1H COSY NMR spectrum of extract CBG (500 MHz, $CDCl_3$)

Supp. Fig. 12. HSQC-DEPT NMR spectrum of extract CBG (500 MHz, $CDCl_3$)

Supp. Fig. 13. HMBC NMR spectrum of extract CBG (500 MHz, $CDCl_3$)

Supplementary Material 2: U(H)PLC-MS/MS parameters

Supplementary Material 3: reference IC50 profiles and literature values

.

Supplementary Material 1: NMR spectra

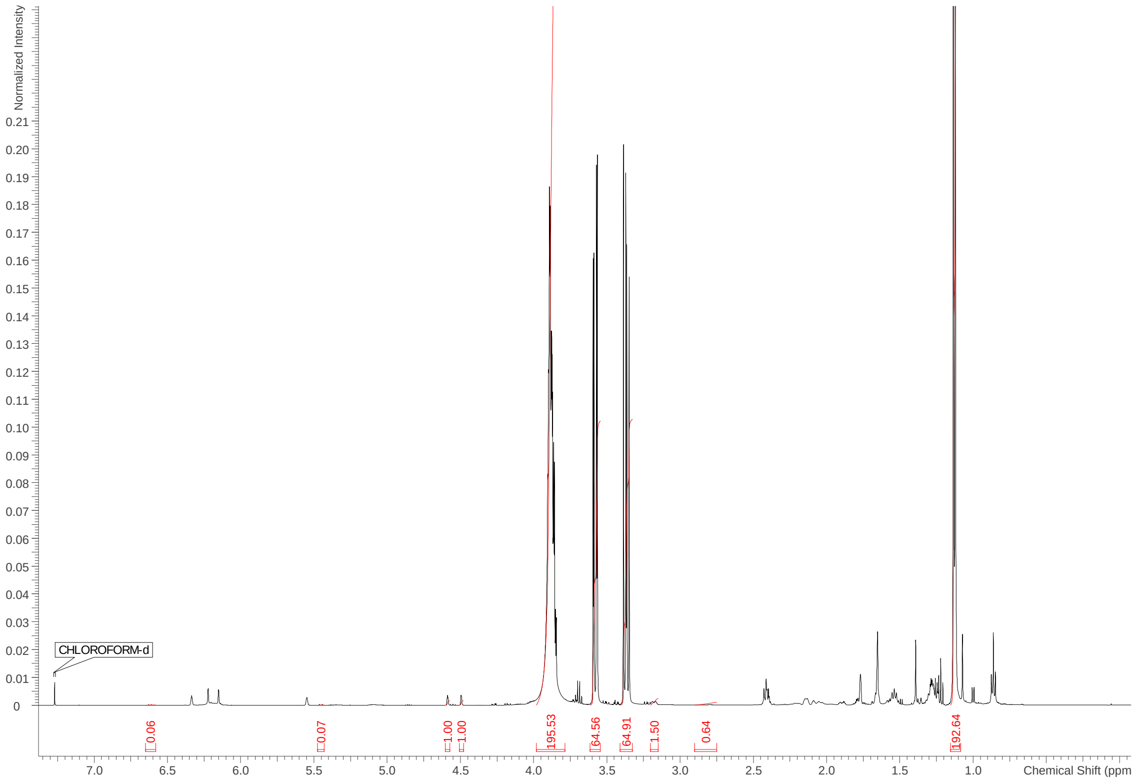

Supp. Fig. *1. ^{1}H NMR spectrum of extract nabiximols (500 MHz, $CDCl_3$) with integrals used for relative quantification.*

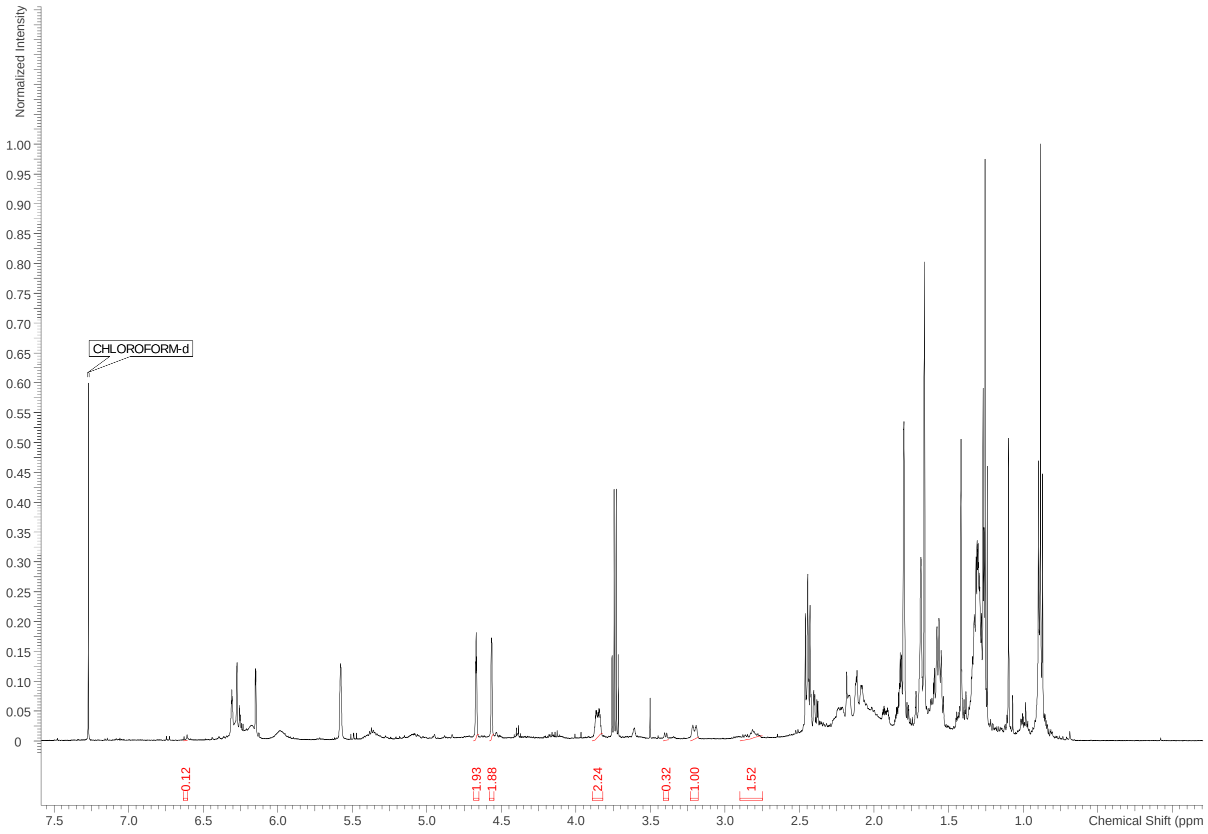

Supp. Fig. *2. ¹H NMR spectrum of extract THC-CBD (500 MHz, $CDCl_3$) with integrals used for relative quantification.*

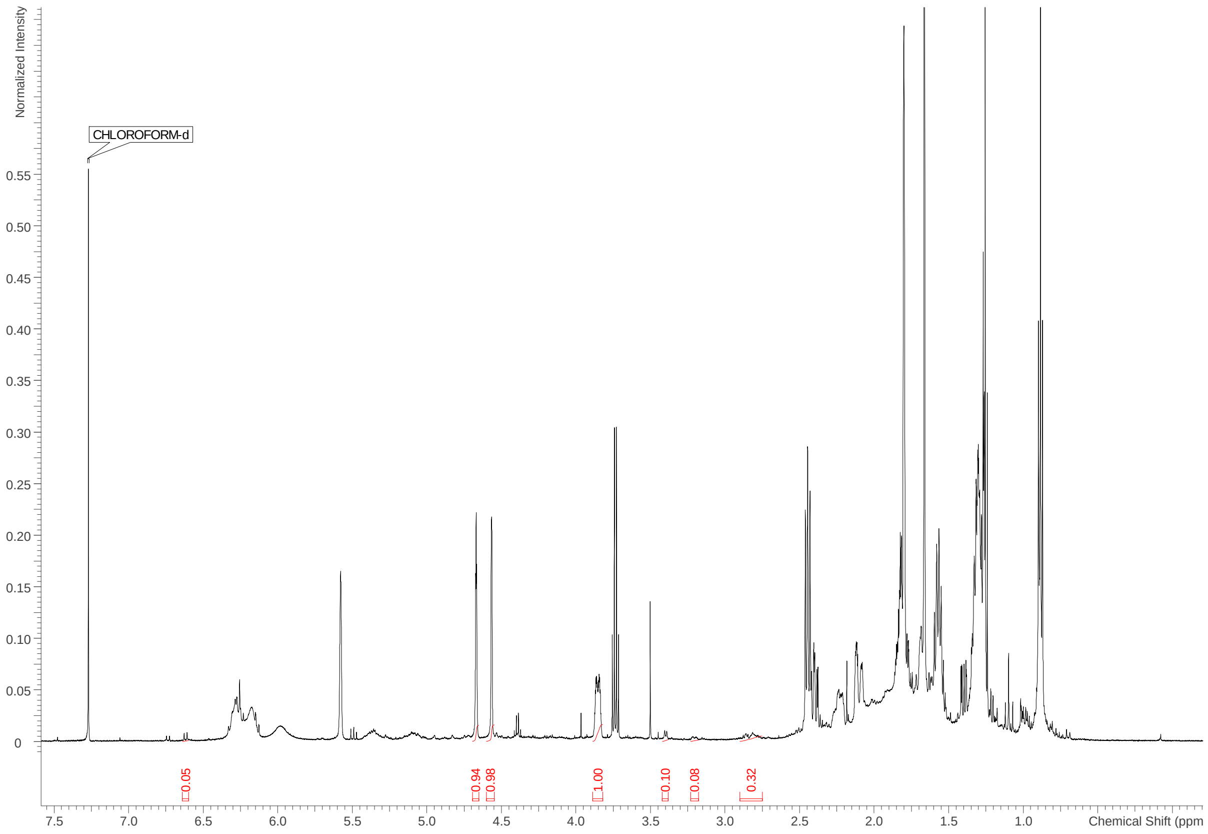

Supp. Fig. *3. ¹H NMR spectrum of extract CBD (500 MHz, $CDCl_3$) with integrals used for relative quantification.*

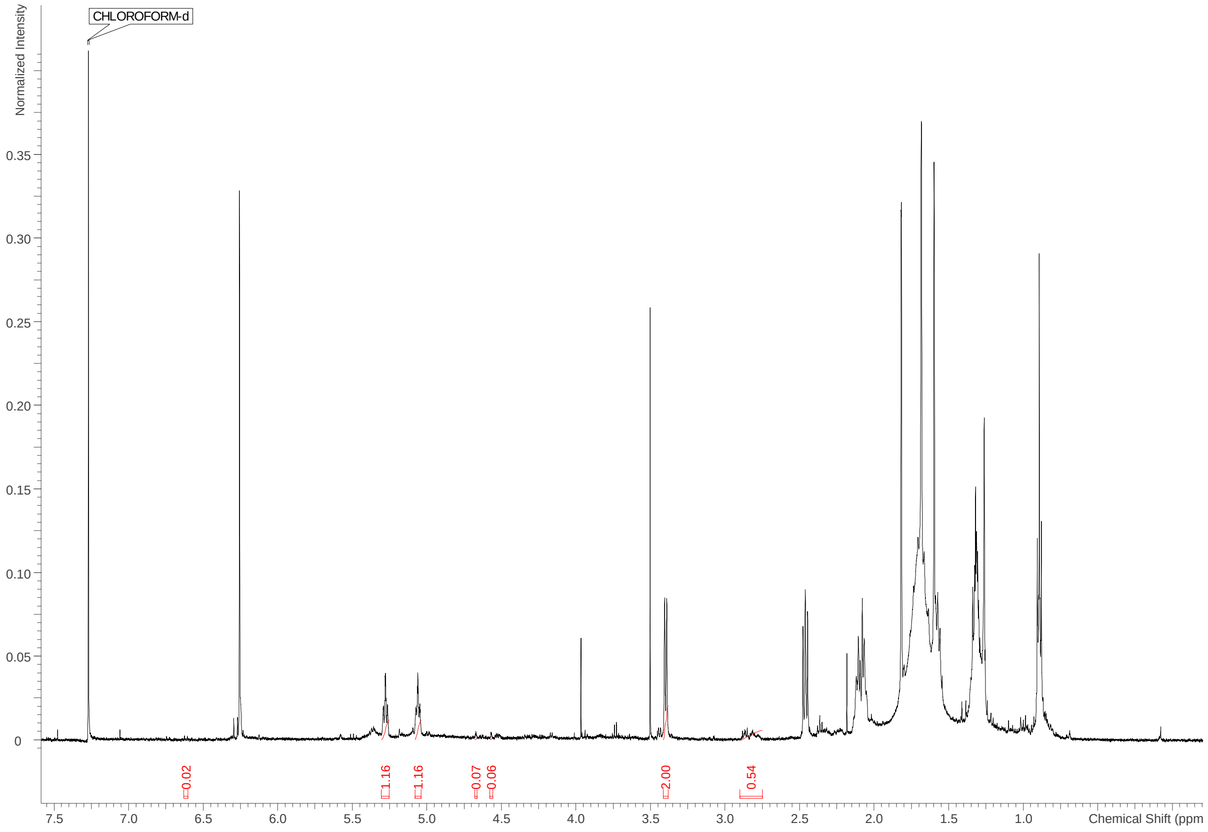

Supp. Fig. *4. ¹H NMR spectrum of extract CBG (500 MHz, CDCl₃) with integrals used for relative quantification.*

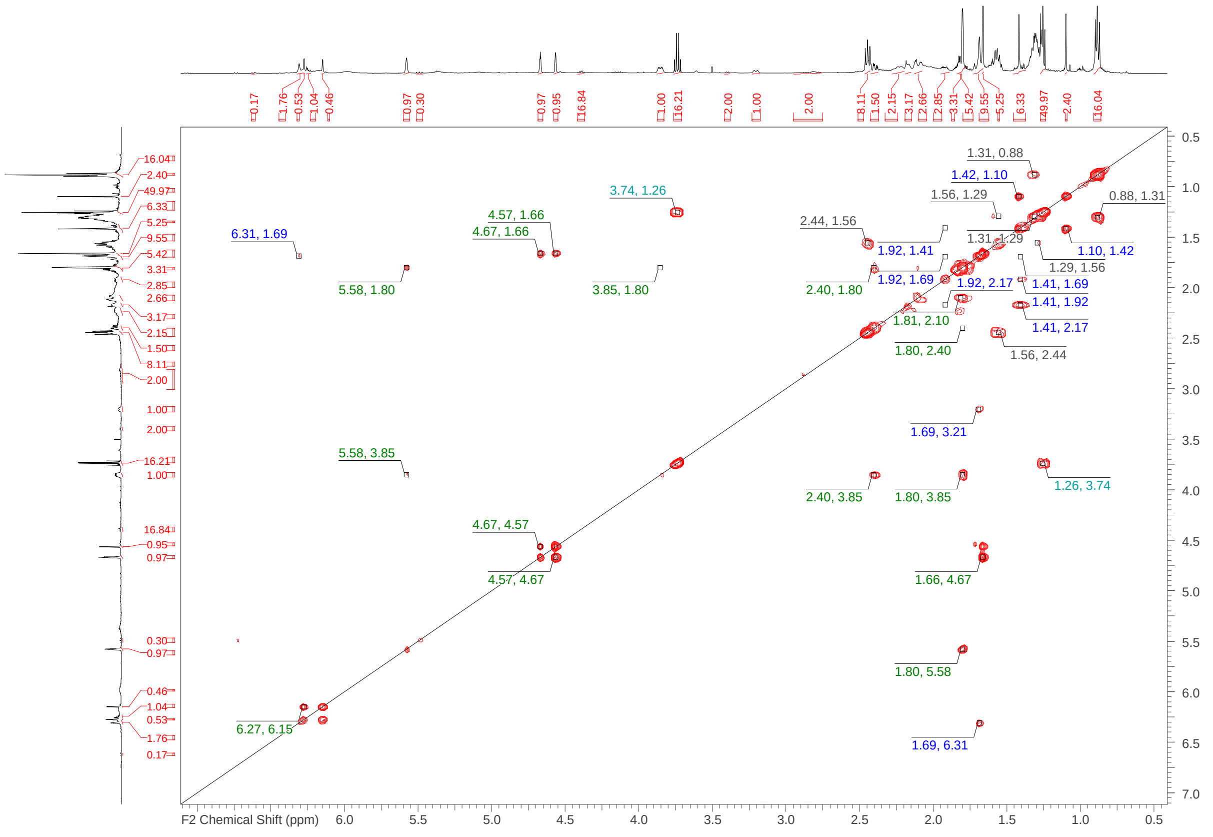

Supp. Fig. *5. ¹H-¹H COSY NMR spectrum of extract THC-CBD (500 MHz, CDCl₃).*

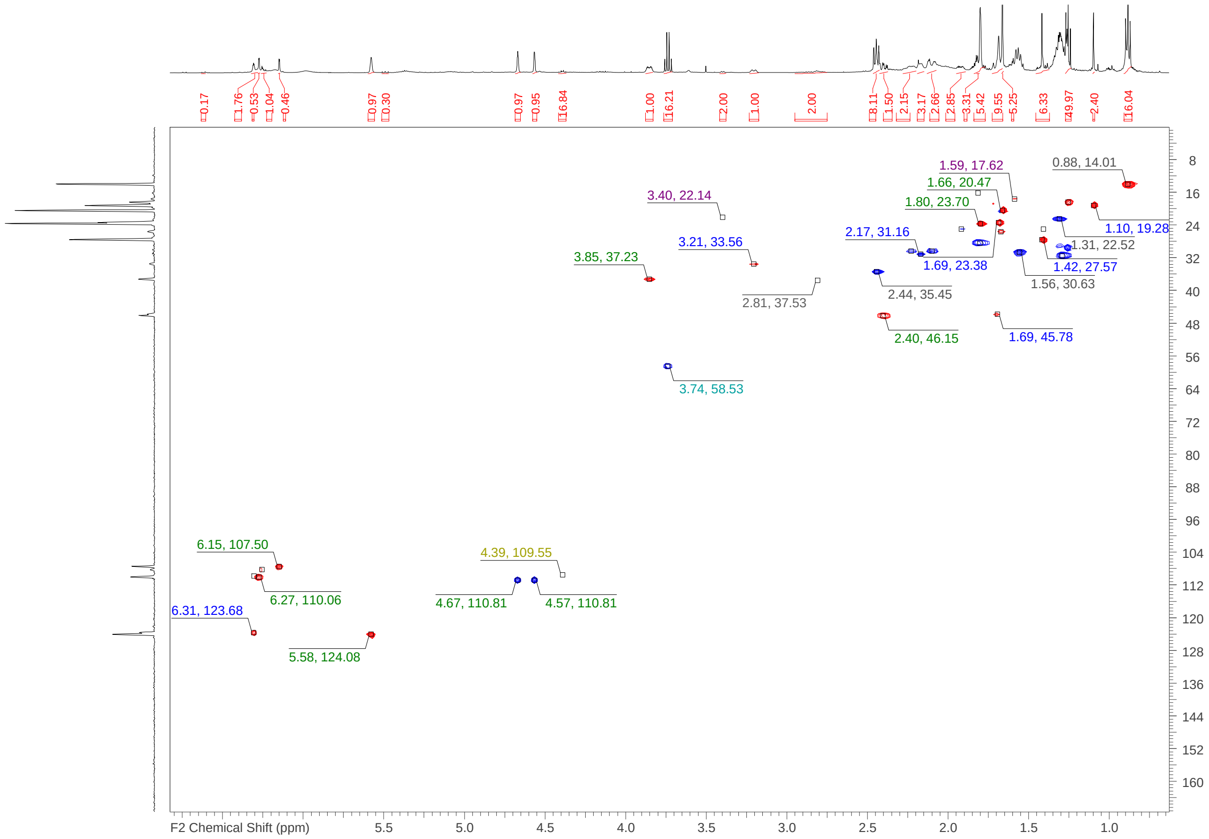

Supp. Fig. *6. HSQC-DEPT NMR spectrum of extract THC-CBD (500 MHz, CDCl₃).*

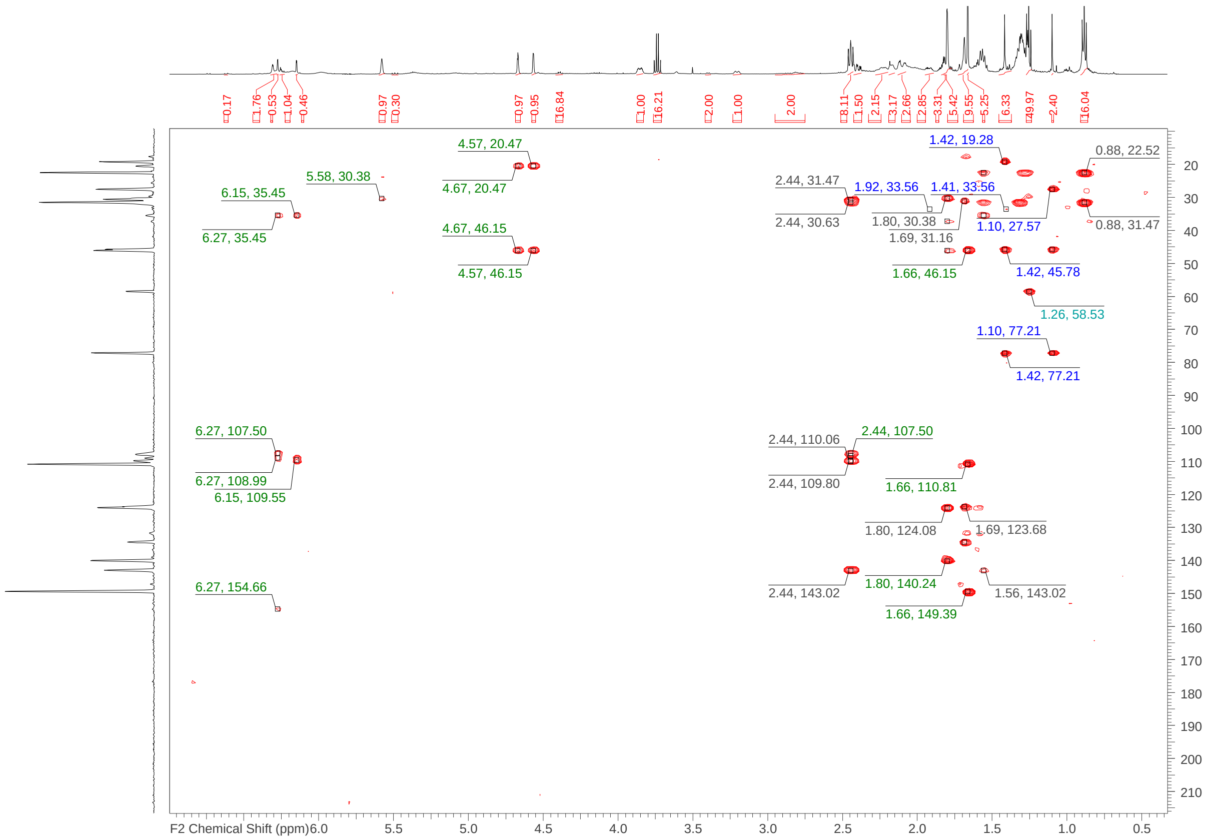

Supp. Fig. *7. HMBC NMR spectrum of extract THC-CBD (500 MHz, CDCl₃).*

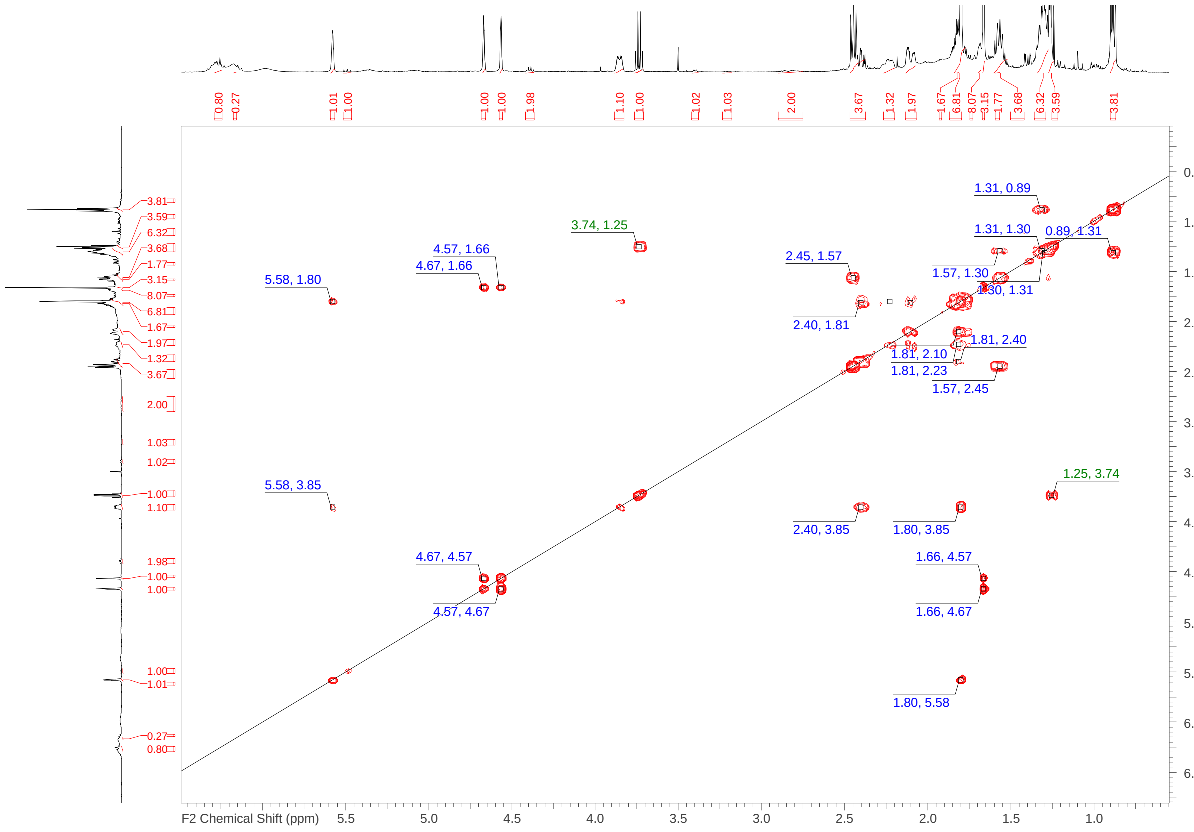

Supp. Fig. 8. *^{1}H-^{1}H COSY NMR spectrum of extract CBD (500 MHz, $CDCl_3$).*

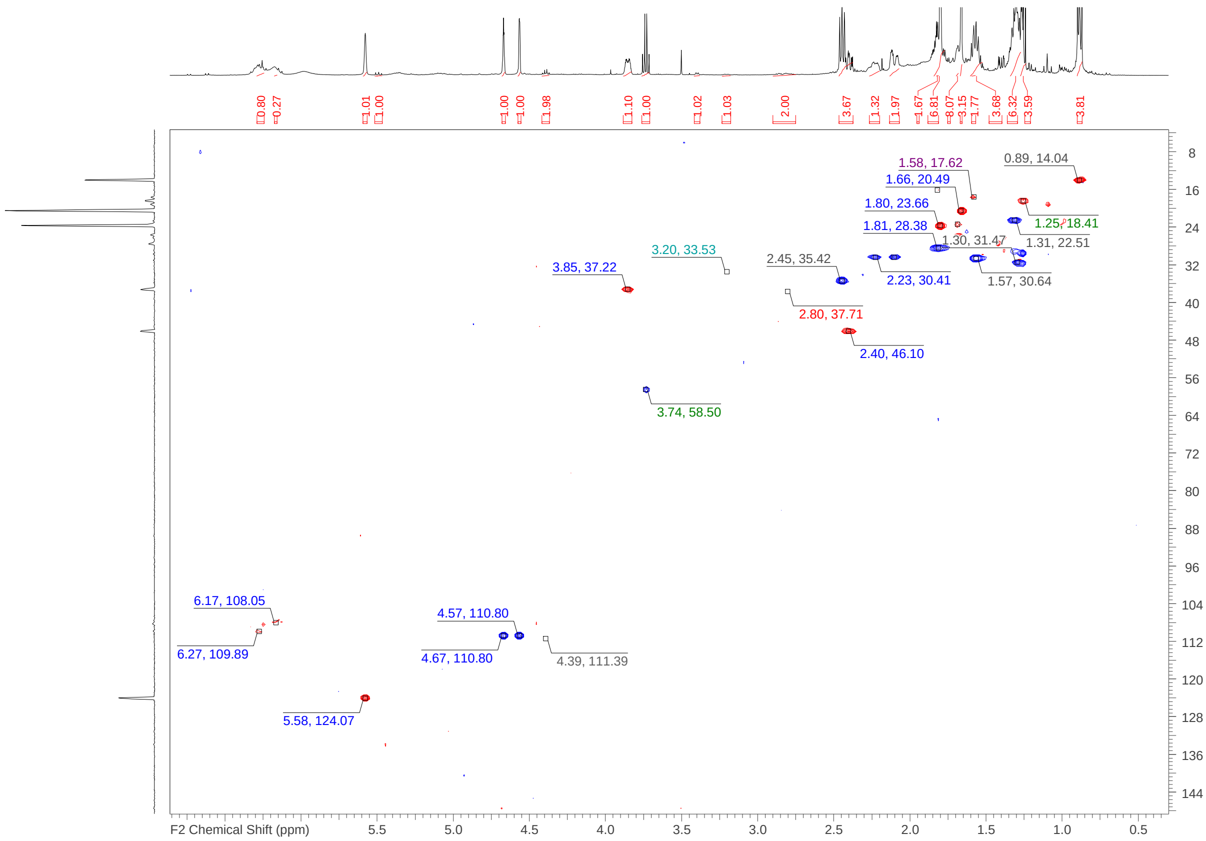

Supp. Fig. 9. *HSQC-DEPT NMR spectrum of extract CBD (500 MHz, $CDCl_3$).*

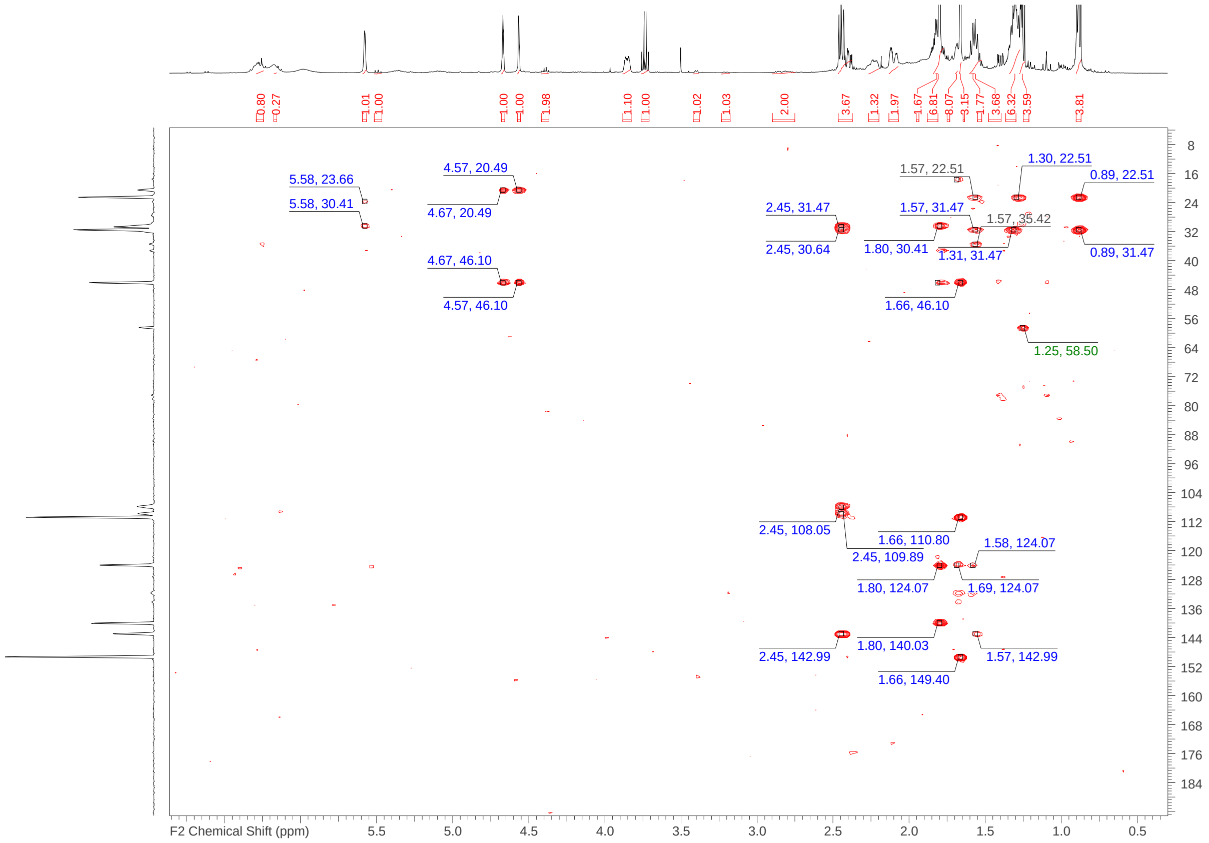

Supp. Fig. *10. HMBC NMR spectrum of extract CBD (500 MHz, $CDCl_3$).*

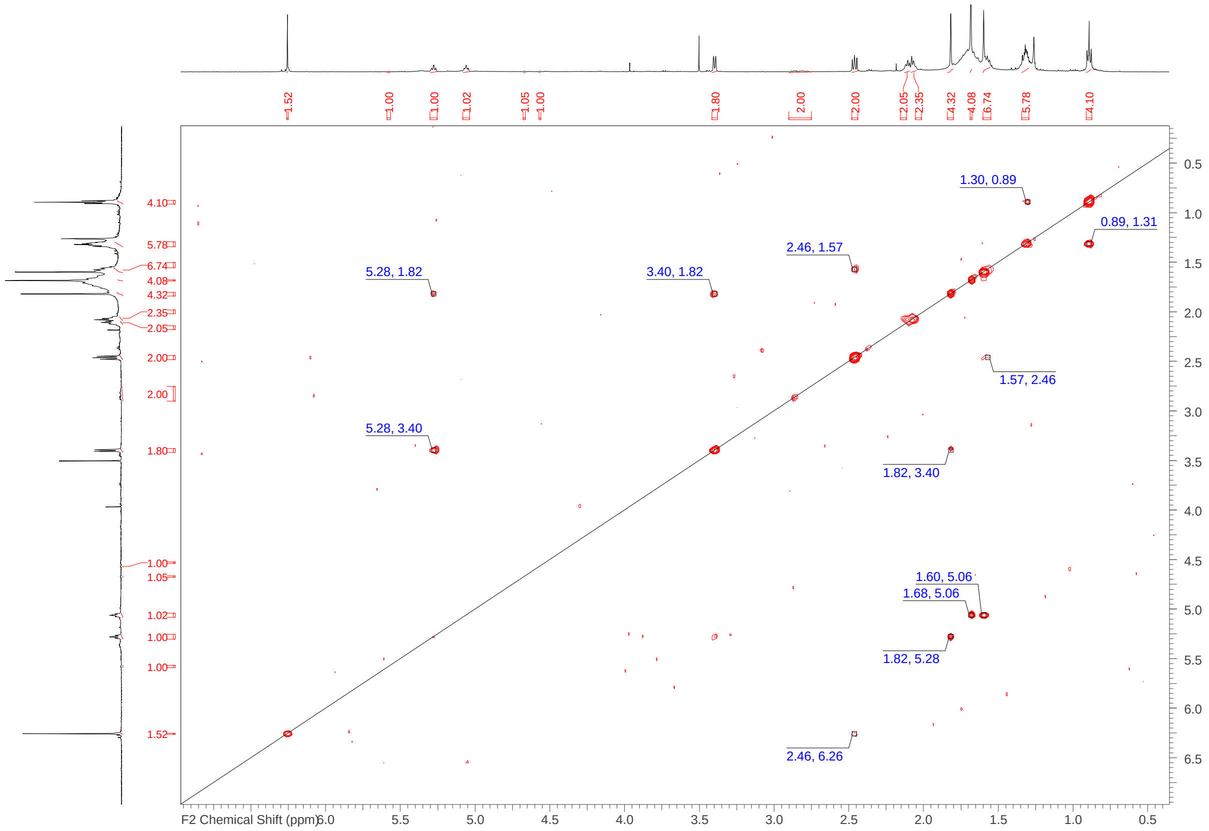

Supp. Fig. *11.. ^1H-^1H COSY NMR spectrum of extract CBG (500 MHz, $CDCl_3$).*

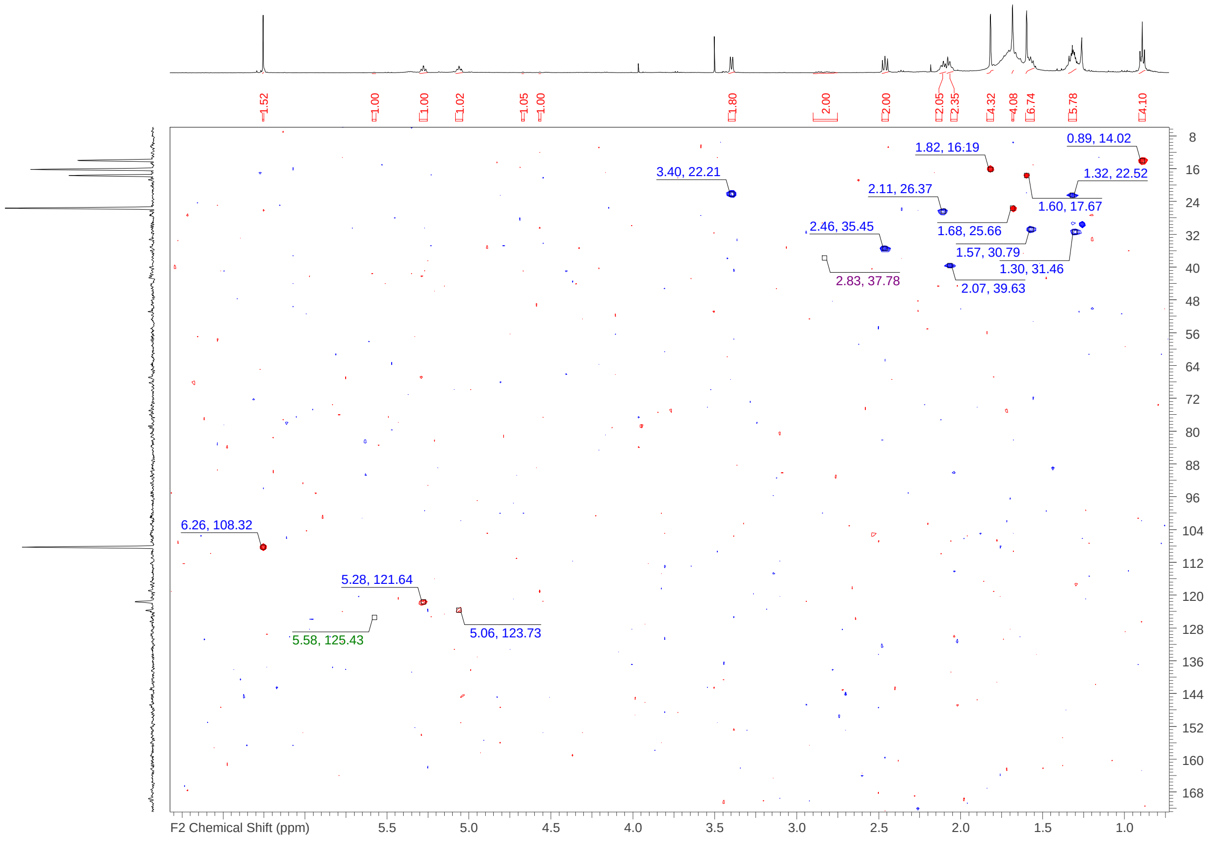

Supp. Fig. *12. HSQC-DEPT NMR spectrum of extract CBG (500 MHz, $CDCl_3$).*

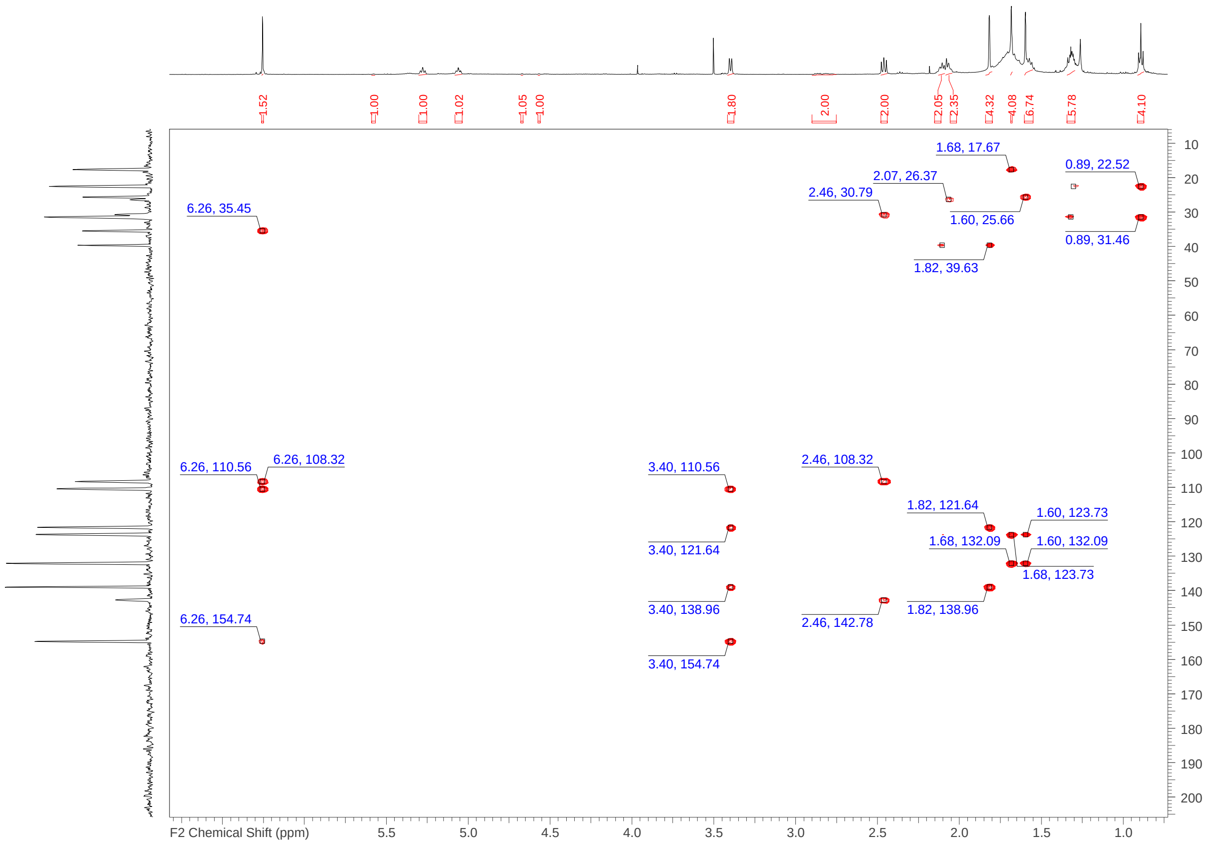

Supp. Fig. *13. HMBC NMR spectrum of extract CBG (500 MHz, $CDCl_3$).*

Supplementary Material 2: U(H)PLC-MS/MS parameters

Instrument: Agilent 6460 Triple Quadrupole MS system, 1290 Infinity LC system (binary capillary pump G422A, column oven G1316C, multisampler G7167B).

Column: Acquity UPLC HSS T3 column, 100 mm x 2.1 mm, 1.8 µm, Waters Corp.

Mobile phase A1: MilliQ water containing 5 % acetonitrile and 0.1 % formic acid

Mobile phase A2: MilliQ water containing 5 % acetonitrile, 0.05 % formic acid and 5 mM ammonium formate

Mobile phase B1: Acetonitrile containing 0.1 % formic acid

Mobile Phase B2: Acetonitrile containing 0.05 % formic acid

Time schedule (%B): 0 – 0.5 min: 0%, 0.5 – 5 min: linear gradient up to 100%, 5 – 5.5 min: 100%, 5.5 – 6 min: 0%.

Autosampler temperature: 10°C

Column temperature: 45°C

Test analytes:

**Compound**	**Q1 m/z**	**Q3 m/z**	**CV**	**CE**	**Mobile Phases**
Warfarin	309.1	163.0, 65.1	92	10, 74	A1B1
Phenprocoumon	281.1	203.0, 175.0	117	10, 22	A2B2
Acenocoumarol	354.1	163.0, 296.0	112	10, 14	A1B1
Testosterone	289.2	97.0, 109.0	137	18, 22	A1B1
Tolbutamide	271.1	91.0, 74.1	87	30, 10	A1B1

Internal standards:

**Internal standard**	**Q1 m/z**	**Q3 m/z**	**CV**	**CE**	**Mobile Phases**
Warfarin D5	314.2	163.0	92	10	A1B1
Phenprocoumon D5	286.2	203.0	112	10	A2B2
Acenocoumarol D4	358.1	167.1	102	14	A1B1
Testosterone D3	292.2	97.0	137	22	A1B1
Tolbutamide D9	280.2	83.1	87	10	A1B1

Reference metabolites (pure analytes):

**Metabolite**	**Q1 m/z**	**Q3 m/z**	**CV**	**CE**	**Mobile Phases**
7-OH-Warfarin	325.1	179.0, 267.0	77	10, 14	A1B1
7-OH-Phenprocoumon	297.1	175.0, 203.0	57	22, 10	A2B2
7-OH-Acenocoumarol	370.1	179.0, 312.0	102	10, 18	A1B1
7-OH-Testosterone	305.2	269.1, 91.1	97	10, 66	A1B1
4-OH-Tolbutamide	287.1	89.0, 74.1	77	42, 10	A1B1

CV = cone voltage, CE= collision energy

Supplementary Material 3: reference IC50 curves and literature values

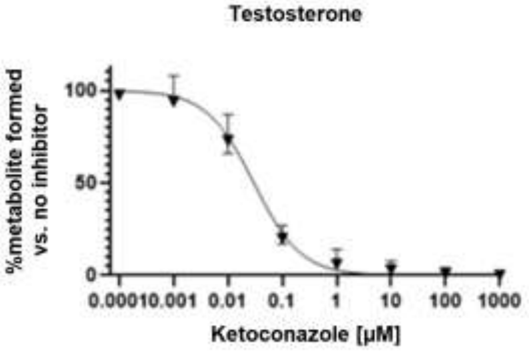

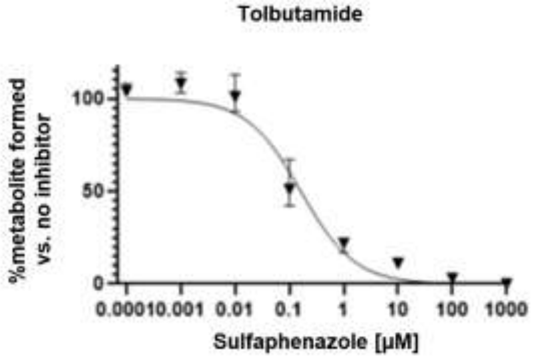

Left: IC_{50} curve obtained in test system using specific CYP3A4 inhibitor ketoconazole and specific CYP3A4 substrate testosterone. Right: IC_{50} curve obtained in test system using specific CYP2C9 inhibitor sulfaphenazole and specific CYP2C9 substrate tolbutamide

Inhibitor	Substrate	IC_{50} (µM pure compounds, µg/ml extracts) [95% CI]	Literature IC_{50} or *K_i (µM) in pooled HLM	Reference PMID
Sulfaphenazole	Tolbutamide	0.16 [0.10-0.19]	0.14-1.3	9491822, 23946123
Extract THC-CBD	Tolbutamide	2.4 [1.2-4.8]		
Extract Nabiximols	Tolbutamide	1.7 [0.9-3.2]		
Extract CBD	Tolbutamide	0.12 [0.06-0.23]		
Extract CBG	Tolbutamide	0.074 [0.032-0.167]		
CBD (pure)	Tolbutamide	0.76 [0.43-1.35]		
THC (pure)	Tolbutamide	0.081 [0.040-0.19]		
Ketoconazole	Testosterone	0.032 [0.023-0.038]	0.045-0.056	12814972, 23729559
Extract THC-CBD	Testosterone	3.0 [1.6-5.4]		
Extract Nabiximols	Testosterone	5.5 [3.2-9.5]		
Extract CBD	Testosterone	0.55 [0.25-1.2]		
Extract CBG	Testosterone	0.63 [0.26-1.5]		
CBD (pure)	Testosterone	2.5 [1.4-4.6]	3.16 ± 0.96	32587099
THC (pure)	Testosterone	23.6 [8.9-68.6]	26.09 ± 6.78	32587099
Extract THC-CBD	Acenocoumarol	22.3 [13.7-36.8]		
Extract Nabiximols	Acenocoumarol	31.3 [21.3-48.9]		
CBD (pure)	Acenocoumarol	1335 [843-2654]		
THC (pure)	Acenocoumarol	73.3 [40.7-135.3]		
Sulfaphenazole	Acenocoumarol	2.5 [1.3-4.6]	*0.5-1.3	8220911, 11038154
Extract THC-CBD	Warfarin	0.55 [0.30-0.85]		
Extract Nabiximols	Warfarin	4.2 [2.6-6.9]		
CBD (pure)	Warfarin	47.0 [24.8-88.5]	*5.6	21356216
THC (pure)	Warfarin	6.9 [4.7-10.5]	*1.5	21356216
Sulfaphenazole	Warfarin	0.35 [0.20-0.62]	0.232-0.246	25757926
Extract THC-CBD	Phenprocoumon	NC		
Extract Nabiximols	Phenprocoumon	NC		
CBD (pure)	Phenprocoumon	NC		
THD (pure)	Phenprocoumon	NC		
Sulfaphenazole	Phenprocoumon	NC	n.a.	

NC = not calculated, n.a. = not available, PMID = PubMed identifier
